# Vocal acoustic characteristics in pre-school aged children

**DOI:** 10.1016/S1808-8694(15)31099-5

**Published:** 2015-10-19

**Authors:** Viviane Michele Cappellari, Carla Aparecida Cielo

**Affiliations:** 1 Master's degree in Human Communication Disorders - UFSM, Speech therapist; 2 Doctor, Full professor in the graduate program on Human Communication Disorders, Santa Maria Federal University. Santa Maria Federal University

**Keywords:** acoustic analysis, children, speech therapy, voice

## Abstract

Evaluation is the first step for any treatment. Therefore, normal parameters are the bases for proper evaluation.

**Aim:**

Verify measures and vocal acoustic characteristics of 23 pre-school aged children of both genders, aged four to six years and eight months.

**Methods:**

The sampling process comprised a questionnaire -that was sent to parents, auditory screening and vocal-perception auditory assessment, based on the R.A.S.A.T. scale. Acoustic analysis was carried out through the Multi Dimensional Voice Program.

**Study:**

Prospective and cross-sectional.

**Results:**

The noise-harmonic ratio (NHR) and frequency variation (vf0) of the total sample was higher than what was found for five and six-year-olds. As age increased, NHR values decreased. As the total mean of amplitude perturbation quotient (APQ) increased, vf0, variation of amplitude (vAm) soft phonation index (SPI) and NHR also increased; the same occurred between mean total NHR and vf0. As the total means of pitch perturbation quotient (PPQ) and voice turbulence index (VTI) increased, SPI mean value decreased.

**Conclusions:**

The results of the acoustic parameters at the age of four years seem to show an immaturity of the structures and lack of neuro-muscular control at that age and the maturation process onset happens near five and six years old.

## INTRODUCTION

Various studies have been conducted for studying voice to establish normalcy levels for voice assessment indices. Many of these articles aimed to establish acoustic parameters for the infant voice, as this form of evaluation may be effective and especially non-invasive, which is a major advantage in children.

The acoustic analysis is an evaluation method that has not been part of the clinical routine of most speech therapists, but which has helped significantly in establishing normalcy parameters, given its objectivity and the possibility of storing data for later analyses and comparisons.

This study aimed to offer a contribution in the description of voice patterns in children; more specifically, the purpose was to check the acoustic voice features of male and female children aged between 4 and 6 years, all of whom were pre-school students in public and private schools in the city of Porto Alegre, RS.

## MATERIAL AND METHOD

### Research subjects

The sample population included male and female children aged between 4 and 6 years and eight months, all of whom were pre=school students in public and private schools in the city of Porto Alegre, RS. Inclusion criteria were a normal or adequate voice and with no reports of airway infection, altered auditory thresholds, a previous history of neurological,^1^ psychiatric or gastric diseases, singing in choirs or as soloists, having had previous phonoaudiological treatment or having undergone previous laryngeal surgery.

### Ethical issues

Verbal explanations about ethical issues and research procedures were given to school directors, parents and caretakers during the first contact with four public and private pre-schools that agreed to participate. The director of each school received and signed an Institutional Authorization Term form, and parents received and signed a Free Informed Consent form, both of which contained the same information that had been given orally before. Both forms were based on the CONEP 196/96 guidelines, and were duly approved - as was the study project - by the Research Ethics Committee of the Santa Maria Federal University (protocol number 099/05).

## MATERIAL

The material for the sample selection process was composed of a questionnaire based on the literature, an Amplivox model A260 audiometer, a TK Missouri otoscope, a Creative model Muvo TX FM (USB 2.0, 256MB, coupled microphone) digital voice recorder, the NHC Swift Sound Wave Pad v. 3.05 audio editing software running on a PC Pentium 266HHZ, CD-ROM, 16 MB RAM computer, and the Multi Dimensional Voice Program - Key Elemetrics acoustic analysis software.

## PROCEDURES

### Sample selection process

A total 118 free informed consent forms were distributed to parents of children in the intended age group for this study in four schools. Of these, one parent or caretaker did not authorize his child to participate, and 10 parents did not return the forms. Two of the children that were authorized to participate were transferred to another school. The sample selection process, therefore, was started with 104 children; their parents not only authorized their participation, but also answered a questionnaire about the general health status of each child. All of the children underwent auditory screening and a perceptual auditory evaluation of voice as part of the sample selection process.

Auditory screening was done at 1000, 2000 and 4000Hz, according to the literature;^2^ only children with normal auditory thresholds were sent to the next stage.

There were 83 children with normal auditory thresholds. These children were sent to the last stage of the sample selection process, which consisted of recording a voice sample for the perceptual auditory evaluation of voice. In this process, each subject was asked to stand with arms held along the body; the recorder microphone was placed 4cm from the subject's mouth.^3^^,^^4^

In this sample selection stage, five of the 83 subjects did not attend school during the sample voice collecting process. The final sample contained the voice samples of 78 subjects, which were sent to four referees (speech therapists with a master's degree or master's degree graduate students.^5^

The R.A.S.A.T^6^ scale, which is an adaptation into Portuguese of the G.R.B.A.S. scale developed by the Japanese Laryngology Society,^7^ was used in the perceptual auditory analysis. The R.A.S.A.T. scale assesses hoarseness (R), roughness (A), soprosity (S), asteny (A) and stress (T), which are graded individually as: 0 - normal, 1 - mild change, 2 - moderate change, and 3 - severe change.

### Acoustic analysis

Following the perceptual auditory analysis by the referees, 23 children with adequate voices were selected, of whom 7 were aged between four years and four years eleven months (n=7), 11 were aged between five years and five years eleven months (n=11), and 5 were aged between six years and six years eight months (n=5).

The voice sample for acoustic analysis was the same that had been recorded for the perceptual auditory analysis, which included sustaining the vowel /a/ for at least three seconds.^4^^,^^8^^,^^9^ The acoustic analysis software extracted the following acoustic parameters of the voice signal: the fundamental frequency (f0), the noise-harmonic ratio (NHR) the pitch perturbation quotient (PPQ), the amplitude perturbation quotient (APQ), the voice turbulence index (VTI), the soft phonation index (SPI), the frequency variation (vf0), and the amplitude variation (vAm), which are described in the literature as those most frequently used in speech therapy.^4^^,^^10^, ^11^, ^12^

### Data analysis

Results were grouped by parameter and age range; however, a normal interval based on the literature was only possible for analysis of the fundamental frequency (f0), as normative data for the remaining parameters are lacking. The maximum and minimum means for establishing the normal fundamental frequency intervals for each age group were: age four, male – 266.6Hz to 375Hz; age four, female – 285.6 to 355Hz; age five, male 247.5 to 350Hz; age five, female 247.6 to 355Hz; age six, male 247.4 to 325Hz; age six, female 247.0 to 315Hz.^13^^,^^14^^,^^15^^,^^16^^,^^17^^,^^18^^,^^19^^,^^20^^,^^21^

### Statistical treatment

Descriptive statistics, Student's t test, Pearson's correlation analysis, and the analysis of variance (ANOVA) were used for the statistical analysis. Spearman's coefficient was used for evaluating the reliability of the evaluation of referees.

As the literature has reported that there may be organic disease in subjects with voices considered as normal, we repeated the statistical analysis with 80% of the sample to increase the degree of reliability of the perceptual auditory analysis. In other words, 20% of the total sample was randomly removed after which the same statistical tests were reapplied.

## RESULTS

Data were tabulated and the results were analyzed according to the total sample (TS) and separately for each age group. The statistical significance was noted, comparing the means for each age group and the mean of the TS. Pearson's coefficient was used for correlating the variables (acoustic analysis indices and age).

As described above, only f0 was compared with literature data, given the lack of published normative data for the remaining acoustic parameters.

Compared to the literature, only the mean f0 in the four-year age group was slightly below the minimal mean value established in other studies (Table 1); the remaining age groups (five and six years) had means within normal intervals.

**Table 1:** Results per subject for the group mean. minimum and maximum values. and the standard deviation for the f0 at ages four. five and six years.

	Age			Age			Age		
Measurements	years: month	f0 in Hz	Interval *	years: month	f0 in Hz	Interval *	years: month	f0 in Hz	Interval *
	04:00	230.44	below	05:00	237.46	below	06:01	231.1	below
	04:01	255.4	below	05:01	207.15	below	06:01	233.08	below
	04:02	238.79	below	05:02	250.67	within	06:02	300.63	within
	04:04	311.36	within	05:04	298.38	within	06:04	233.33	below
	04:09	267.86	within	05:06	248.45	within	06:08	246.22	below
	04:11	238.97	below	05:06	229.83	below			within
	04:11	242.6	below	05:08	262.05	within			
				05:08	245.65	below			
				05:09	238.32	below			
				05:11	257.88	within			
				05:11	309.1	within			
Mean		255.06	below		253.18	within		248.87	within
Minimum		230.44			207.15			231.1	
Maximum		311.37			309.1			300.63	
Standard deviation		27.75			29.13			29.55	

* Normal interval established based on the mean values in other studies for this age range

The mean index of the frequency variation (vf0) was higher in the four-year age group, although not significantly higher when compared to the other age groups and the TS. The mean vf0 for ages five (p= 0.015) and six (p=0.05) years were significantly higher compared to the TS mean. This results, however, was seen only in the analysis of the TS, not confirmed in the analysis of the reduced sample (RS) (Table 2).

**Table 2:** Means of all parameters that were evaluated per age group and in the total sample (TS).

Parameters	MEAN 4 years	MEAN 5 years	MEAN 6 years	MEAN (AT)	SIGNIFICANCE*
f0 Hz	255.06	253.18	248.87	252.81	–
vf0%	7.01	3.05	2.65	4.17	5 (p=0.015) and 6 years (p=0.05) > AT
vAm %	25.24	31.72	27.72	28.88	
PPQ %	1.21	0.96	0.9	1.02	
APQ %	8.1	5.51	5.98	6.4	
NHR	0.26	0.17	0.17	0.2	4 years (p=0.013) > 5 years
					4 years (p=0.04) > 6 years
					AT (p=0.027) > 5 years
VTI	0.06	0.07	0.06	0.06	
SPI	3.3	4.02	3.25	3.64	

• Statistical analysis using Student's “t,” test and the analysis of variance - ANOVA

• Number value of “p,” ≤ 0.05

* • significant positive correlation

There was a significant difference in the NHR between the means of the four and five-year age groups (p= 0.013) and also between the means of the four and six-year age groups (p=0.04). The comparison of age groups in the

TS revealed a significant difference between the mean for the five-year age group and the TS (p=0.027) (Table 2).

Pearson's coefficient was used for correlating the total means of acoustic variables (f0, vf0, vAm, PPQ, APQ, NRH, VTI, SPI) with each other and with the mean age.

Analysis of the APQ showed a positive statistical correlation between this parameter and three others, the vf0 (p=0.029), the vAm (0.034) and the NHR (p=0.003); this result was found in the analysis of both the TS and the RS.

A negative statistical correlation was found between the mean PPQe of the APQ (p=0.045) and between the PPQ and the SPI (p= 0.021); this result was found in the analysis of both the TS and the RS.

**Table 3:** Correlation between acoustic parameters and between the same and the variable age.

MEASUREMENTS	MEAN	AGE	F0	PPQ	VF0	APQ	VAM	NHR	VTI	SPI
MEAN		4-6 years	252.81	1.02	4.17	6.40	28.88	0.20	0.06	3.64
AGE	4–6 years	–	.757	.312	.098	185	.642	.009**	.639	.861
F0	252.81	.757	–	.734	.731	.450	.339	.837	.721	.540
PPQ	1.02	.312	.734	–	.186	.631	.471	.111	.212	.021**
VF0	4.17	.098	.731	.186	–	.029*	.236	.000*	.790	.107
APQ	6.40	.185	.450	.631	.029*	–	.034*	.003*	.095	.045*
VAM	28.88	.642	.339	.471	.236	.034*	–	.292	.500	.172
NHR	0.20	.009**	.837	.111	.000*	.003*	.292	–	.830	.248
VTI	0.06	.639	.721	.212	.790	.095	.500	.830	–	.029**
SPI	3.64	.861	.540	.021**	.107	.045*	.172	.248	.029**	–

• Statistical analysis using Pearson's correlation • Number value of “p,” ≤ 0.05

* • significant positive correlation

** significant negative correlation

There was a negative statistical correlation between the mean NHR and age (p=0.009). In this same analysis, a positive correlation was found between the total means of the NHR and the vf0 (p= 0.0001). These results were found in the analysis of both the TS and the RS.

A negative correlation was found between the mean VTI for the TS and the total mean SPI (p=0.029); this result was found in the analysis of both the TS and the RS.

## DISCUSSION

Acoustic analysis is a non-invasive evaluation method that makes it possible to separate normal and pathological voice. Its results, however, cannot yet be generalized due to the lack of reference measurements.^18^^,^^20^^,^^22^, ^23^, ^24^, ^25^, ^26^, ^27^ Following a review of studies on acoustic measurements in children, researchers^27^ have found that many papers do not differentiate normal and dysphonic voice sufficiently; this finding underlines a need for further investigation to increase measurement standardization and provide reference values.

In the current study, f0 measurements in the TS and in the five and six year age groups agree with the literature.^14^, ^15^, ^16^, ^17^, ^18^^,^^21^ The four year age group presented a slightly decreased mean, below the minimal and maximal interval established in the literature.^13^, ^14^, ^15^, ^16^, ^17^, ^18^, ^19^, ^20^^,^^28^^,^^29^ This difference, however, was not statistically significant, and does not appear to mean a departure from normalcy.

Our results revealed that as age increased, f0 decreased; there was no significant difference between f0 means in the various age groups when compared to the same means for the TS.

These results are similar to those in other papers^13^^,^^19^^,^^30^ that assessed four to six-year-old subjects with normal voice, which showed that the f0 decreased as age increased.

The f0 is one of the most important measurements of the acoustic analysis; it is directly related with the length, stress, rigidity and mass of the vocal folds, in turn associated with subglottic pressure.^12^ The literature^31^ reports that vocal folds grow 0.4mm each year in females and 0.7mm each year in males until age ^20^.

An increase in the f0 may be more proportional to body growth than to age, suggesting that as children grow, the voice tract also develops; this may have occurred in the subjects of this study.^32^

In studies of children aged five, seven, nine and eleven years, a few authors^33^ have reported that the f0 for males aged five years was significantly higher compared to all the other age groups. Furthermore, the authors reported that there was no statistical difference between the ages seven, nine and eleven years.

Children aged four years in this study had higher vf0 indices compared to the other age groups and to the TS mean, but this difference was not statistically significant.

A comparison of the mean vf0 in each age group with the mean vf0 of the TS revealed a statistically significant difference in favor of the total mean compared to the means of the five and six year age groups. This result is probably due to the fact that the TS mean was increased by the higher value of the four-year age group mean.

Recording the f0 and its standard deviation is the basis for calculating the sustained emission vf0. The vf0 is expected even in normal voice; thus it may be said that sound waves are almost periodic. However, variation beyond expected values may suggest voice diseases or an inability to sustain the emission due to neuromuscular immaturity.^10^^,^^1^

Increased vf0 indices in four-year-olds, which possibly led to an increase in the TS mean, may be explained by the fact that the vocal ligament at this age is still immature; the lamina propria layers and the junction between the vocal ligament and the muscle fibers are not well-defined. Furthermore, at age four years, the mucosa is thinner compared to the newborn, but thicker compared to adults, which reduce the degree of control over vocal folds, and increases instability during phonation.^10^^,^^31^^,^^34^, ^35^, ^36^, ^37^, ^38^

The literature^3^ also states that the mucous layer that lines the vocal folds, under good conditions, allows air to pass through the glottis with no resistance. If the amount of mucus is decreased, however, there may be increased viscosity, resulting in increased friction during the vibratory cycle. If there is more mucus, it may accumulate on the vestibular face of the vocal folds, making them heavier and decreased their vibration ability.

This fact may also be attributed to other variables needed for adequately sustaining voice emission, which may alter the vf0. We found no reports in the literature on the amount or the features of the muco-cilliary layer of vocal folds in children aged four years that might be directly related with an increased vf0 in our sample subjects.

As children grow, their ability to control frequency and intensity also increases, and vocal fold vibratory cycle aperiodicity decreases. This ability results form training and control of voice due to improved neurolaryngological conditions.^37^^,^^39^ Furthermore, vocal fold biomechanical stability, as evidenced by a decrease in the vf0, becomes possible after puberty.^40^

For certain authors^37^^,^^41^ the vf0 suffer interference from jitter and (shimmer), strong indicators of voice instability from cycle to cycle, which are altered according to the phonatory conditions of intensity and frequency.

The measurement of jitter we used in our study was the pitch perturbation quotient (PPQ), which is calculated based on the mean of pitch perturbations from cycle to cycle in the entire voice sample under analysis.^10^^,^^11^^,^^12^^,^^20^^,^^27^

The PPQ results in the current study revealed that this parameter was higher at age four years, although this difference was not statistically significant. These findings agree with those in another study^19^ that found higher jitter levels in the four-year age group compared to the seven-year age group; the author suggested that there was a gradually higher control of emission as age increased, in other words, neurological maturation decreases the aperiodicity of vibratory cycles.^37^

The PPQ indices found in the current study were similar to those reported in the literature,^20^ in which jitter values increased as the f0 increased in children from ages 3 to 10 years.

Other authors^42^ have also found that younger children have higher perturbation of the fundamental frequency, and that as they grow, jitter indices decrease. Furthermore, the authors believe that perturbations of the fundamental frequency are inversely proportional to motor control development,^37^ and that this control is only fully developed at around age 10 years, a finding that converges with those of our study.

A study of 112 normal voiced children^25^ with a diagnosis of papillomatosis, gastroesophageal reflux, atopic disease or vocal nodules, revealed that jitter values increased in the group of subjects with papillomatosis and vocal nodules due to an increased vocal fold mass (which causes irregular vibration and alters vibration regularity from cycle to cycle), in a comparison between groups with pathological and normal voice.

These findings show that an altered mucosa may increase jitter. It may be possible to correlate these findings with the fact that the lamina propria of the mucosa at age four years is quite immature or different from the mucosa at higher ages, which could explain our findings.

Variations in jitter may result from increased vocal fold mass or stress, from the symmetry of structures or from muscle or neural function.^10^

The measurement of shimmer used in the current study was the amplitude perturbation quotient (APQ), which is calculated from the mean of amplitude perturbations cycle after cycle throughout the voice sample.^10^, ^11^, ^12^^,^^20^^,^^27^ Our results for the APQ were higher in children aged four years, although these findings were not statistically significant when compared to the other age groups and to the TS. These results were similar to those found in the literature^20^ in children with a mean age of six years eleven months and no voice disorders. Other studies,^19^ however, have reported that shimmer in their subjects did not increase with age; these studies found that younger children did not have higher shimmer indices.^42^

Researchers have reported wide variations in shimmer and jitter in children, according to sex and age, which also suggest anatomic and physiological changes in laryngeal structures and lack of laryngeal control.^37^^,^^38^

The NHR is considered a measure of noise perturbation that quantifies the portion of noise to the portion of harmonics in a voice sample; it may be very useful for differentiating normal and dysphonic voice.^10^, ^11^, ^12^^,^^27^^,^^43^ The NHR is the mean proportion between the non-harmonic and the harmonic spectra of voice energy.^4^

In the current study, the mean NHR in children aged four years was significantly higher compared to the NHR means at ages five and six years; there was also a significant difference in favor of the total mean compared to the five-year group mean. Additionally, the correlation between age and the total NHR mean was significant; as age increased, the NHR decreased.

The literature has reported^24^ no significant increase in the NHR when comparing groups with pathological voice and groups with normal voice. However, a study^43^ of 46 children with vocal nodules and 31 normal voiced children aged between four and fourteen years, revealed that jitter, shimmer, f0 tremor and NHR measurements were significantly higher in children with nodules compared to normal voiced children.

A study^44^ of 50 boys aged between three and ten years aimed to check the relation between the NHR, the perceptual auditory analysis and the laryngological exam. The results revealed that the NHR was significantly higher in dysphonic boys with structural injury of vocal folds compared to boys with no vocal fold changes.

Most of the studies have described NHR values by comparing normal and pathological voice. Thus, our results showing that the NHR was significantly higher at age four years could not be compared with these findings.^9^^,^^45^, ^46^, ^47^

Based on studies in which the NHR was higher when there was vocal fold injury, it may be seen that mucosal alterations increase the noise component of emissions, which are related - as other authors have suggested - to perceptual auditory noise (hoarseness and roughness). The same reasoning used above in the interpretation of jitter and PPQ results in our study might be applied as follows: the immature mucosa of children aged four years could generate different vibratory conditions, in which there might be more emission noise without necessarily any dysphonia, perceptually and auditorily. Furthermore, an increased NHR at age four years in the current study may be explained, based on the literature,^3^ by the possibility that NHR values results from variations in frequency, amplitude, turbulent noise, sub-harmonic components and emission interruptions.

PPQ values in our sample decreased slightly as age increased, although this reduction was not statistically significant. This may suggest what has been discussed previously: there is more emission stability as the nervous system and structures of children mature and as they acquire more voice experience with age. This possibility is reinforced by the fact that mean PPQ values for ages five and six years were close to those results reported by other authors^44^ who studied children aged between four and fourteen years.

In the current study there was a significant positive correlation between the total mean APQ and the total means of the vf0 and vAm. In other words, as the APQ increases, the vf0 and vAm also increase. This finding is in accordance with the literature^48^ in that instability during voice emission may be classified as long-term and short-term, and may arise singly or in association, generally being due to glottic adduction problems.

The APQ is a measurement of shimmer, which might explain these results, since the vf0 is affected by jitter and shimmer perturbations. These are strong indicators of vocal instability, and may be altered depending on phonatory conditions of intensity and frequency.^41^

As the total mean APQ increased, the mean SPI decreased significantly. This negative correlation with the total mean SPI was also seen in a comparison with the total mean PPQ.

As the total mean NHR increased, the mean vf0 also increased significantly.

The relation between aerodynamic, myoelastic and muco-ondulatory forces for voice production show that there is still expiration airflow during the closure phase of the vibratory cycle. Such flow causes the subglottic pressure, which ends up overcoming the resistance of glottic adduction and initiating a new cycle.^3^^,^^10^^,^^37^

These statements may explain the significant positive correlation between the variables vf0, PPQ, APQ and NHR, all of which may be affected by the lack of adequate neuromuscular control and by transglottic airflow.^37^

Adequate neuromuscular control is needed for maintaining vocal fold firmness and stability so that the vocal folds may resist the air current pressure. There was a significant negative correlation between the SPI and the VTI; as the VTI increased, the SPI decreased, probably because these indices are in opposition. A higher SPI index means that phonation is softer and more fluid (within normal limits). Similarly, a lower VTI index means less turbulence during normal phonation.

In general, most of our findings were in accordance with those in the references that we consulted, reinforcing findings in the Brazilian and international literature and supporting the knowledge that serves as the basis for clinical evaluation in speech therapy and otorhinolaryngology.

## CONCLUSION

The purpose of this study was met based on our results and discussion, enabling us to propose the following points concerning the parameters of acoustic analysis in children aged between four and six years.

The results appear to demonstrate that, at age four years, there is structure immaturity and lack of neuromuscular control, and that ages five and six may be considered as the moment of maturation of phonatory structures.

Our study reached mean results for each acoustic parameter according to the age groups we investigated; we were thus able to propose standard measurements for normal infant voices, data not found in the literature that we consulted. These results need to be supported by similar studies with larger samples for these measurements to attain statistical power. Given the current lack of such studies, this paper may provide support for voice assessments in children aged four to six years.
